# Correlation Between Vitamin D Deficiency and Diabetic Ketoacidosis

**DOI:** 10.7759/cureus.4497

**Published:** 2019-04-18

**Authors:** Aqsa Iqbal, Abid Hussain, Anum Iqbal, Vinesh Kumar

**Affiliations:** 1 Cardiology, University of Illinois at Chicago, Chicago, USA; 2 Internal Medicine, Nishtar Medical College, Multan, PAK; 3 Internal Medicine, Liaquat University of Medical and Health Sciences, Hyderabad, PAK; 4 Internal Medicine, Ghulam Mohammad Mahar Medical College, Sukkur, PAK

**Keywords:** diabetic ketoacidosis, type 1 diabetes, vitamin d levels

## Abstract

Both type 1 and type 2 diabetes mellitus have been associated with vitamin D deficiency. Diabetic ketoacidosis, which is a complication of type 1 and, rarely, type 2 diabetes, is also found to be associated with vitamin D levels. This review discusses studies on the correlation between diabetic ketoacidosis and vitamin D levels. Studies show that vitamin D deficiency is associated with the occurrence of diabetic ketoacidosis. Diabetic ketoacidosis is also found to affect vitamin D levels. The possible explanation of diabetic ketoacidosis affecting vitamin D levels is the inactivity of the 1-alpha-hydroxylase enzyme and an increase in the renal excretion of vitamin D binding proteins. The presence of vitamin D receptors on pancreatic beta cells explains the role of vitamin D in the causation of diabetic ketoacidosis.

## Introduction and background

Vitamin D deficiency is prevalent around the globe, with an estimated one billion people being vitamin D deficient [1]. The relationship between vitamin D deficiency and type 1 and type 2 diabetes mellitus has long been established. Several studies on animals as well as on humans have supported the notion that both type 1 and type 2 diabetes mellitus are associated with vitamin D deficiency [2-5]. Furthermore, supplementation of vitamin D reduces the risk of type 1 diabetes [6]. Diabetic ketoacidosis is a complication of uncontrolled type 1 diabetes mellitus, which results from insulin deficiency or excess of adrenaline or cortisol [7]. Additionally, diabetic ketoacidosis can also occur in patients with type 2 diabetes. The incidence rate of diabetic ketoacidosis is 0-263 per 1000 per year, whereas the prevalence of diabetic ketoacidosis is 1-128/1000 per year [8]. A few studies demonstrated the association of vitamin D levels with the occurrence and severity of diabetic ketoacidosis [9-12]. This paper discusses the relationship of vitamin D levels with diabetic ketoacidosis in light of the current literature. The objective of this review is to find out the correlation of vitamin D levels with diabetic ketoacidosis so that vitamin D supplementation can become an integral part of diabetic ketoacidosis prevention and management. This review also provides direction for future research.

## Review

From our search of PubMed, Web of Science, Google Scholar, and Embase, we found four observational studies and one case report on the association of diabetic ketoacidosis with vitamin D levels. The characteristics of the observational studies are mentioned in Table 1.

**Table 1:** Characteristics of studies on the correlation between diabetic ketoacidosis and vitamin D levels

Study design	Population (mean age in years)	Results	Year	Country	Author
Observational	Children and adolescents with type 1 diabetes mellitus (8.6 years)	Diabetic ketoacidosis (DKA) contributes to 25 (OH) vitamin D3 deficiency in children with type 1 diabetes and vice versa	2009	Australia	Hyunh T [9]
Observational	Children and adolescents with type 1 diabetes mellitus (8.7 years)	Low bicarbonate levels were significantly associated with low 25 (OH) vitamin D3 levels	2013	India	Devidayal [10]
Observational	Children with type 1 diabetes (N/A)	Low 25 (OH) vitaminD3 levels in children with diabetic ketoacidosis (DKA)	2013	Kurdistan	Gheini [12]
Observational	Children with type 1 diabetes (8.7 years)	Low 25 (OH) vitamin D3 levels were seen to be worse in type 1 diabetic children with low bicarbonate levels as compared to type 1 diabetes children with normal bicarbonate levels *	2016	USA	Al-Zubeidi [11]

Overview of previous studies

According to our search, the first study on this topic was conducted by Hyunh et al. in 2009. In that study, Hyunh observed that 85% of type 1 diabetic children with low vitamin D levels had low bicarbonate levels of less than 18 ng/ml and 50% of patients with diabetic ketoacidosis (DKA) had vitamin D deficiency. They also found that with the correction of acidosis, vitamin D levels normalized in 83.3% of children, which shows a strong relationship of vitamin D levels with diabetic ketoacidosis. However, the sample size in their study was very small. They started with 110 children with type 1 diabetes. Vitamin D data of only 64 children were available. Vitamin D deficiency was present in 14 children. In total, 28 children were diagnosed with diabetic ketoacidosis by bicarbonate levels and pH, and 12 children with diabetic ketoacidosis had vitamin D deficiency. With the correction of acidosis, vitamin D levels normalized in 10 of the 11 children, with one child lost to follow-up. In addition to the small sample size, they did not consider many factors affecting vitamin D levels after the correction of ketoacidosis. The follow-up period of vitamin D levels assessment in individuals after the correction of acidosis was different, which could have caused bias. Although there was some potential bias in the study, it provides a statistically significant correlation between low vitamin D levels and diabetic ketoacidosis. The study also suggests that while interpreting vitamin D levels in patients with diabetes, acid-base status should be assessed and attention should be paid toward the correction of vitamin D levels [9]. Table 2 summarizes the number of participants in the observational studies included in this review.

**Table 2:** Number of participants in studies on the correlation between diabetic ketoacidosis and vitamin D levels

Author	Total participants	Participants with vitamin D levels	Participants with low bicarbonate levels	Participants with both low vitamin D and bicarbonate levels
Hyunh T [9]	64	14	28	12
Devidayal [10]	32	32	32	32
Gheini [12]	-	-	21	20
Al-Zubeidi [11]	185	107	61	44

In a study conducted in India by Devidayal et al. in 2013, they observed the correlation between bicarbonate levels and vitamin D levels in 32 type 1 diabetic children with the age range between one to 12 years. They found a positive correlation between bicarbonate levels and 25(OH) vitamin D levels at the time of the diagnosis of diabetic ketoacidosis and after a month. However, vitamin D levels were not corrected after the normalization of bicarbonate levels. Besides, bicarbonate levels in the body mass index of diabetic ketoacidosis patients was another factor that affected vitamin D levels at follow-up. Notably, there was no significant association between pH and vitamin D levels in this study, which could be explained by tachypnea in diabetic ketoacidosis patients [10].

Al-Zubeidi conducted an observational study in 185 new-onset type 1 diabetic patients [11]. They found a strong association between vitamin D levels and diabetic ketoacidosis. Vitamin D levels were low in type 1 diabetic patients in this study and were even lower in patients who presented with diabetic ketoacidosis. There was a 5 ng/ml average increase of vitamin D levels with the resolution of acidosis. Vitamin D-deficient (VDD) individuals were more likely to be in diabetic ketoacidosis than vitamin D-sufficient (VDS) individuals. They also found a relationship between age and vitamin D deficiency. Individuals with diabetic ketoacidosis and age less than 10 years were more likely to be vitamin D insufficient individuals, which was defined according to Endocrine Society Guidelines. Improvement in 25(OH) vitamin levels was seen three days post diabetic ketoacidosis. The prevalence of vitamin D deficiency in diabetic ketoacidosis was more than half. The average increase of 5ng/ ml of vitamin D levels was seen after correction of acidosis [11].

Gheini et al. conducted a study in Kurdistan that also showed a correlation of vitamin D levels with diabetic ketoacidosis. Due to language constraints, we cannot comment on the overall methodology and all the results; however, this study demonstrated low vitamin D levels in 95% of type 1 diabetic children with diabetic ketoacidosis, which offers further evidence of this association [12].

A case report supported the correlation of functional vitamin D deficiency with diabetic ketoacidosis. The case report discussed a 10-year-old girl who developed diabetic ketoacidosis as an initial presentation of Type II vitamin D-dependent rickets (VDDR type II). Although vitamin D levels were normal, diabetic ketoacidosis in the case depicts the role of vitamin D in the prevention of diabetic ketoacidosis [13].

Diabetic ketoacidosis due to vitamin D deficiency

Acidosis in diabetic ketoacidosis patients might be due to the contribution of vitamin D in insulin secretion and improving insulin sensitivity. Vitamin D is well studied to have a role in insulin secretion and insulin sensitivity. Low levels of insulin are seen in mice with either vitamin D deficiency or non-functioning vitamin D receptors [14]. Additionally, the replacement of vitamin D improved insulin secretion. The stimulatory effect of vitamin D on insulin is due to an increase in calcium.

Parathyroid hormone (PTH) levels rise with vitamin D deficiency and increased parathyroid hormone levels have been evidenced to reduced insulin sensitivity. Studies have shown that vitamin D deficiency cause insulin insensitivity and insulin sensitivity improved with vitamin D supplementation [15-16]. Rudnicki et al. demonstrated in their study that intravenous administration of vitamin D lowered insulin levels in women with gestational diabetes mellitus, which infers the improvement of insulin sensitivity [17]. The sample size was very small in the study, which could have caused bias.

Vitamin D is also known to protect against viral and bacterial infection, which are precipitating factors for diabetic ketoacidosis [18]. Vitamin D deficiency is studied to cause metabolic acidosis. The absorption of bicarbonate from proximal renal tubules reduces with vitamin D deficiency [19].

Role of acidosis in causing low vitamin D levels

Low vitamin D levels have been associated with metabolic acidosis for a long time. Acidosis seems to affect vitamin D levels in patients with diabetes more than without diabetes. A greater change in vitamin D levels was seen after the correction of acidosis in patients with diabetes. The correction of acidosis showed the mean difference of 5 ng/ml in vitamin D levels. In almost all studies that have been done so far on the correlation of vitamin D levels with diabetic ketoacidosis, bicarbonate levels correlated more strongly with vitamin D levels than pH [9].

Interestingly, vitamin D levels were normalized in type 1 diabetes children after the correction of diabetic ketoacidosis. Furthermore, low vitamin D levels contributed to the incidence of diabetic ketoacidosis in patients with type 1 diabetes [10].

The impact of metabolic acidosis on 1, 25 (OH) Vitamin D level is known from both human and animal studies over the years. Reddy et al. conducted a study on mice in which he induced metabolic acidosis in mice by feeding them aqueous ammonium chloride for nine days. He found a significant negative correlation between activated vitamin D levels (1, 25 (OH) Vitamin D) and hydrogen ion concentration [20].

Acidosis hinders the activation of the vitamin D by inactivating 1-alpha-hydroxylase enzyme, which lowers the conversion of 25 (OH) vitamin D3 to 1, 25 (OH) vitamin D3. It has also been seen to decrease the vitamin D binding protein, which reduces 25 (OH) vitamin D3 correspondingly [21].

Recommendation

We recommend that until more studies are produced, vitamin D should routinely be checked in patients with diabetes. In the case of low vitamin D levels, vitamin D should be supplemented to avoid the risk of diabetic ketoacidosis.

Direction for future research

More studies need to be done to study the relationship of vitamin D deficiency with diabetic ketoacidosis. Studies should be done in different regions around the globe and with a larger sample size to reach consensus and minimize the bias. While designing studies, all factors that affect vitamin D levels and increase the risk of diabetic ketoacidosis should be kept in mind. Additionally, liquid chromatography-tandem mass spectrometry should be used as a gold standard for measuring vitamin D levels in future studies [22].

Factors that affect vitamin D levels are high altitude, seasons, time of day, clothing, atmospheric component, sunscreen use, skin pigmentation, high body mass index (BMI), kidney diseases, and chronic illnesses, whereas factors that precipitate diabetic ketoacidosis are [23-24].

1) Younger age (highest risk in an age of fewer than two years)

2) Ethnic minority

3) Lack of health insurance

4) Low body mass index (BMI)

5) Preceding infection

6) Delayed management of blood sugar

This review led us to the following questions:

1) Should vitamin D supplementation become an integral part of diabetic ketoacidosis management?

2) What dose of vitamin D is required to prevent diabetic ketoacidosis?

3) What is the actual mechanism behind vitamin D deficiency due to diabetic ketoacidosis?

## Conclusions

From studies included in this review article, it is concluded that vitamin D deficiency and diabetic ketoacidosis are interrelated. Diabetic ketoacidosis was found to decrease vitamin D levels, which, in turn, contributed to the occurrence of diabetic ketoacidosis. These findings suggest the examination of vitamin D levels in patients with diabetes and the administration of vitamin D supplements to patients with deficiencies in order to prevent the risk of diabetic ketoacidosis. Although all studies we found through our database search show a positive correlation between vitamin D deficiency and diabetic ketoacidosis, more clinical research is required to make a conclusive correlation.
